# Deficits in a Simple Visual Go/No-go Discrimination Task in Two Mouse Models of Huntington’s Disease

**DOI:** 10.1371/currents.hd.fe74c94bdd446a0470f6f905a30b5dd1

**Published:** 2013-11-07

**Authors:** Stephen Oakeshott, Andrew Farrar, Russell Port, Jane Cummins-Sutphen, Jason Berger, Judy Watson-Johnson, Sylvie Ramboz, David Howland, Dani Brunner

**Affiliations:** PsychoGenics Inc., Tarrytown, New York, USA; PsychoGenics Inc., Tarrytown, New York, USAPsychoGenics Inc.; PsychoGenics Inc., Tarrytown, New York, USA; PsychoGenics Inc., Tarrytown, New York, USA; PsychoGenics Inc., Tarrytown, New York, USA; PsychoGenics Inc., Tarrytown, New York, USA; PsychoGenics Inc., Tarrytown, New York, USA; CHDI Foundation Inc, Princeton, New Jersey, USA; PsychoGenics Inc., Tarrytown, New York, USA; Columbia University, New York, New York, USA

## Abstract

Huntington’s disease (HD), a devastating neurodegenerative disorder caused by a CAG repeat expansion on the HTT gene located on chromosome 4, is associated with a characteristic pattern of progressive cognitive dysfunction known to involve early deficits in executive function. A modified Go/No-go successive discrimination task was designed to assess the type of online response control/executive function known to be disrupted in patients with HD. The present studies show that this simple discrimination assay revealed early and robust deficits in two mouse models of HD, the zQ175 KI mouse (deficits from 28 weeks of age) and the R6/2 mouse, carrying ~240 CAG repeats (deficits from 9 weeks of age). These deficits are not due to gross motor dysfunction in the test animals, but instead appear to measure some inability to inhibit responding in the HD mouse models, suggesting this assay may measure deficits in underlying attentional and/or behavioral inhibition processes. Accordingly, this assay may be well suited to evaluation of simple deficits in cognitive function in mouse HD models, providing a potential platform for preclinical screening.

## Introduction

Huntington’s disease (HD) is a dominant autosomal disorder resulting from expansion of a stretch of CAG repeats near the N-terminus of the HTT gene that encodes for the protein huntingtin. Since the discovery of this gene mutation in 1993 1 many mouse models of the disease have been generated for use in preclinical screening with the aim of providing a platform to evaluate potential therapies. At present HD is invariably fatal, with the interval from the presentation of initial symptoms to death typically being around 15 to 20 years 7. Clinically, HD is characterized by a triad of symptom groups with progressive psychiatric, cognitive, and motor dysfunction, with the non-motor aspects of the disease generally held to cause a significant burden to patients and caregivers even relatively early in disease progression 8.

The discovery of mutant huntingtin has yielded the advent of predictive genetic testing, allowing examination of the prodromal phase of HD. Accordingly, increasing emphasis has been placed on understanding the neurobiological underpinnings of early cognitive symptoms in HD, both in humans carrying the mutation and mouse models of the disease. One domain of cognitive performance which is known to be affected early in HD is executive function 9 ^,^ 10, broadly characterized as a set of processes underlying behavioral flexibility and control of responses. Patients are reported to have deficits on laboratory tasks such as the Wisconsin Card Sorting Task, requiring a shift of attention from one set of features to another, or in problem solving tasks like the Tower of London which require them to plan a sequence of actions 11 ^,^ 12, coupled with difficulties in their daily lives associated with multi-tasking or planning, even to the extent of difficulty with simultaneously walking and talking 8.

In parallel, recent efforts have begun to evaluate analogous cognitive functions in genetically modified mouse models of HD, demonstrating deficits in behavioral flexibility 13 ^,^ 14 ^,^ 15. Additional studies have employed operant testing to evaluate cognitive and behavioral abnormalities across different mouse models of HD 16 ^,^ 17, (see 18 for review). The present experiments sought to assess executive function in different mouse models of HD using a modified Go/No-go successive discrimination task, in which animals were trained to make a response to gain food reinforcement (Go condition) and subsequently to withhold responding in the presence or absence of a discriminative stimulus (No-go condition). It was hypothesized that mouse models of HD would be impaired in their ability to discriminate the Go and No go stimulus conditions relative to wild type control animals. This pattern of dysfunction is thought to stem from progressive neurodegenerative alterations to frontal-striatal circuitry, with regions of the striatum known to be amongst the earliest sites of neurodegeneration in HD.

## Materials and Methods

**Subjects**

***R6/2 CAG 240 model (CHDI-81001005)***

Initial testing was carried out using the R6/2 CAG 240 mouse, a widely studied transgenic fragment model carrying a small section of the N-terminus of the mutant human *HTT* gene 4. This mouse model is known to demonstrate both cognitive 13 and motor 19 ^,^ 20 deficits from an early age.

R6/2 CAG 240 mice were bred in-house at PsychoGenics Inc., by crossing transgenic R6/2 CAG 240 males with wild type (WT) females, all on a congenic C57Bl6J background. A small cohort of male mice (6 per genotype) was evaluated in this initial study, with a mean CAG repeat length of 247.5 repeats, ranging from 245 to 249 repeats. At weaning, test subjects were single-housed in standard mouse cages, maintained on a 12:12 light cycle with free access to water. All testing was conducted during the light phase of the light/dark cycle. Subjects were maintained at 85% of their ad libitum body weight, factoring in expected growth, by daily feeding of limited quantities of food (BIO-SERV 500mg pellets). The animals tested were 45 days old ± 5 days at the start of initial training and were 62 days old ± 5 days at the start of discrimination training.

***R6/2 CAG 240 F1 (CHDI-004-(1)(6)) model*** **

R6/2 CAG 240 mice on a C57Bl/6J x CBA/CaJ F1 background, along with littermate controls, were bred in our facility by crossing ovarian transplant C57Bl/6J female animals with WT CBA/CaJ males. In this study, animals were weaned into paired housing conditions in opti-MICE cages (Animal Care Systems, CO), remaining in these groupings throughout the experiments. A cohort of 8 mice per sex per genotype was evaluated, with a mean CAG repeat length of 234 (ranging from 228 to 247) in the R6/2 CAG 240 F1 ****animals. Subjects were maintained at 85% of their ad libitum body weight, factoring in expected growth, by daily feeding of limited quantities of food (BIO-SERV 500mg pellets). The animals were all 45-46 days old on the initial day of pre-training and ranged from 66 to 71 days of age on the initial day of discrimination training.

For both R6/2 mouse lines, this test age was selected because R6/2 CAG 240 F1 mice exhibit a progressive disease phenotype with mortality beginning at approximately 18 weeks of age (Menalled et al., 2009).

***BAC HD F1 (CHDI-81001012) model***

BAC HD F1 mice, carrying ~97 stable CAGCAA repeats on a C57Bl/6J x FVB/NJ F1 background were bred at the Jackson Laboratory (Bar Harbor, ME) and shipped to the test facility as adults. Mice were maintained throughout the experiment in opti-MICE cages (Animal Care Systems, CO) on a 12:12 light cycle with free access to water. Once acclimated to the colony, the animals were pair-housed and food restricted, with WT mice reduced to 85% of their free-feeding body weights (one WT animal was single housed during the course of the experiment, as the cage mate died prior to the start of testing).

The BAC HD F1 mice, which develop excess fat deposits, were food-restricted gradually to the point where a matched control group consumed comparable amounts of food to 85% WT animals in a 30-min free feeding test. At this point, the BAC HD F1 were slightly heavier than WT mice (mean 36.5 g vs. 28.1 g on the initial day of discrimination training). A single group of female mice was evaluated, consisting of 11 WT and 10 BAC HD F1 animals, with the animals 81 weeks of age ± 1 week at the start of discrimination training. This test age was selected because we have previously observed cognitive deficits in this mouse line at a similar age 21. The mice had previously been evaluated in the PhenoCube, an automated phenotyping system, but were naïve to operant boxes and had never before been food restricted.

***zQ175 KI (CHDI-81003003) model at 74 weeks of age***

Testing was also carried out in the zQ175 KI CAG 175 mouse, a novel knock-in model generated at PsychoGenics, Inc., (Tarrytown, NY; see 5 for a detailed description of this line), derived from the CAG 140 mouse 6. This mouse line was tested to confirm that the phenotype observed is not specific to the R6/2 CAG 240 and BAC HD mouse lines and to further generalize these findings to a model that exhibits more subtle impairment and employs a genetic construct that more closely resembles the human condition.

Homozygous (homo), heterozygous (het) and WT knock-in (KI) mice were bred in our facility by crossing pairs of zQ175 KI het mice, all on a congenic C57Bl/6J background. The mice were maintained throughout experimentation in opti-MICE cages (Animal Care Systems, CO) on a 12:12 light cycle with free access to water. Over the course of this testing, all mice were single-housed and kept at 85% of their ad libitum body weight by daily feeding of limited quantities of food (BIO-SERV 500mg pellets).

The test ages for the zQ175 line described below were selected to assess the progression of severity in cognitive deficits, beginning at an age in which motor deficits are relatively mild 5.

A small cohort of male mice was evaluated in this experiment (5 WT, 6 het and 4 homo mice). Prior to this testing, these animals underwent a comprehensive behavioral test battery, involving a variety of motor and cognitive tests, but were naïve to operant boxes and had never before been food-restricted. At the start of pre-training, all animals were 74 weeks old and were 75 weeks old at the start of discrimination training. The het mice carried a mean of 192.8 CAG repeats (ranging from 187 to 198 repeats), while the homo animals carried a mean of 185.8 repeats (ranging from 171 to 194). During discrimination training, one het animal had to be removed from the study due to poor health, with all data from this animal excluded.

***zQ175 KI (CHDI-81003003) model at 54 weeks of age*** **

A mixed sex cohort was evaluated in this experiment, with a total of 11 WT mice (5 male and 6 female), 11 zQ175 KI het mice (6 male and 5 female) and 10 zQ175 KI homo (5 male and 5 female) tested. These animals had previously undergone a comprehensive behavioral test battery, involving a variety of motor and cognitive tests, but were naïve to operant boxes and had never before been food-restricted. The zQ175 KI het mice in this cohort carried a mean CAG repeat length of 186.8 repeats (ranging from 176 to 203), while the zQ175 KI homo mice had a mean CAG repeat length of 187.1 (ranging from 183 to 192 repeats). Operant pre-training began when the mice were 54 weeks of age, with discrimination training beginning with the animals aged 56 ± 1 weeks.

***zQ175 KI (CHDI-81003003) model at 26 weeks of age*** **

A cohort of het and WT KI mice were bred in our facility by crossing zQ175 KI het male animals with WT females, all on a congenic C57Bl6J background. The mice were single housed in opti-MICE cages (Animal Care Systems, CO) on a 12:12 light cycle with free access to water, while they were kept at 85% of their ad libitum body weight by daily feeding of limited quantities of food (BIO-SERV 500mg pellets).

A mixed sex cohort of 24 mice was evaluated in this experiment, evenly split into 6 mice per sex per genotype. Prior to the present experiment, these animals had been evaluated in the PhenoCube, a comprehensive behavioral screening platform, and the tapered beam motor assay, but were naïve to operant boxes and had never before been food restricted. At the start of pre-training, all mice were 26 weeks old and were 28 ± 1 weeks old at the start of discrimination training. The het mice in this study carried a mean of 192.9 CAG repeats, ranging from 187 to 205 repeats.

**Equipment**

R6/2 CAG 240 mice were tested in standard mouse operant chambers (Med Associates, VT), measuring approximately 16 cm long x 14 cm wide and 13 cm high walls, whereas all other mice were testing in slightly larger chambers measuring 22 cm long x 18 cm wide, with 13 cm high walls. Otherwise, the operant chambers were configured identically in all experiments. Each chamber contained a nosepoke recess, which could be illuminated by a small embedded light-emitting diode (LED), located centrally on the wall opposite the food magazine. The boxes also contained two retractable levers, one on either side of the food magazine, but these were not employed in the current experiments. The chambers were located within individual sound attenuating shells, with a fan mounted at one end of the sound-attenuating cubicle which was active throughout behavioral sessions. Reinforcement was provided by time-limited access to a dipper containing evaporated milk (Carnation™, OH). The hardware was controlled and all events were recorded by the Med-PC IV software package.

**Behavioral procedures – pre-training**

Following food restriction and two days of magazine training, all animals were trained to nosepoke via a simple free operant procedure, where the nosepoke recess was active throughout a 40 min session and nosepoking was reinforced with 4 s access to an evaporated milk reinforcer on a response-initiated fixed-interval 20 s (FI20) schedule. The illumination of the nosepoke recess and houselight (light or dark) was counterbalanced across animals in each genotype, with the lights either on or off throughout the session. No reinforcement was delivered without a nosepoke. Animals were trained to a criterion, requiring them to obtain 40 reinforcers across 2 consecutive sessions. Training was carried out daily Monday to Friday, with the animals resting over the weekends, and continued until all test mice had reached the criterion. Following criterion achievement, R6/2 CAG 240 mice received a single session of training with a response-initiated variable-interval 30 s (VI30) schedule, whereas mice in all other studies received one further session of FI20 training. Mice that did not acquire the performance criterion within 15 FI20 sessions were excluded from subsequent discrimination testing.

BAC HD F1 mice had previously been trained in a separate procedure, requiring them to nosepoke for food reinforcement on a fixed ratio 1 (FR1) schedule in a lit nosepoke recess. Consequently, it was not possible to counterbalance the group as in the other experiments, such that all these animals were trained to nosepoke to a lit recess. After completion of the previous experiment, these mice rested in their home cages for two weeks, then received two days of training on the same FI20 procedure described above, at which point all mice passed the acquisition criterion. Discrimination training then proceeded as described below.

**Behavioral procedures – discrimination training**

Discrimination training sessions followed completion of instrumental pre-training. These sessions were also 40 min in duration, presenting the animals with both potentially reinforced and unreinforced periods, with the availability of reinforcement signaled by the illumination state of the nosepoke recess (the houselight was not used during this phase). In all cases, the light condition presented in pre-training served as the reinforced state, such that the animals were required to learn to avoid responding in the novel condition. No other source of illumination was utilized during discrimination training. The sessions were unbalanced, with 30 min of potentially reinforced time presented pseudorandomly in blocks of 30, 60, 90, 120 or 150 s, interspersed with 10 min of unreinforced time presented pseudorandomly in blocks of 10, 20, 30 or 60 s. Nosepoking was reinforced during the potentially reinforced periods on a response-initiated VI5 schedule with 3 s of access to the milk reinforcer.

Discrimination performance was indexed by a discrimination ratio calculated by dividing the response rate in the reinforced condition by the sum of the response rates in the reinforced and the unreinforced conditions for each mouse for each session.

All studies were carried out in strict accordance with the recommendations in the Guide for the Care and Use of Laboratory Animals, NRC 2011. The protocol was approved by the Institutional Animal Care and Use Committee of PsychoGenics, Inc. (PHS OLAW animal welfare assurance number A4471-01), an AAALAC International accredited institution (Unit #001213).

**Statistical methods**

Discrimination ratio and response rate data were evaluated using repeated measures analysis carried out with SAS (SAS Institute Inc.) using Mixed Effect Models, based on likelihood estimation. The models were fitted using the procedure PROC MIXED 22. Genotype and session, and their interactions were considered in all the models, with sex and light/dark training condition evaluated separately where possible. Significant interactions were followed up with analyses of simple main effects. Because we were particularly interested in the differences in response rates between genotypes when discrimination performance was more stable, average response rates from the final four days of discrimination training were analyzed separately with planned comparisons. Analyses of pre-training data were carried out with *t*-tests or one-way analysis of variance using Statview (SAS Institute Inc.). An effect was considered significant if *P*< 0.05.

## Results

**R6/2 CAG 240 model**

Pre-training

Only 4 mice per genotype progressed to discrimination training in this study due to failure to achieve the performance criterion within 15 test sessions, leaving a final sample size per genotype of 4 animals. Amongst these mice, there were no differences in their rate of instrumental acquisition, with WT mice requiring an average of 3.8 days to reach criterion while R6/2 CAG 240 mice required an average of 4.0 days to reach criterion (*t*_(6)_ = 0.24, *P* > 0.80).

Discrimination training

The R6/2 CAG 240 mice were significantly impaired in acquisition of this task (Fig. 1A). A two-way ANOVA (Genotype x Session; Light/Dark Condition was not included as a factor due to the small number of mice participating in the experiment) revealed a significant overall effect of Genotype (F_(1,6)_ = 38.70, *P* < 0.001), along with a significant interaction between Genotype and Session (F_(11,66)_ = 3.32, *P* < 0.01), but no overall effect of Session (F_(11,66)_ = 1.21, *P* > 0.25). Follow-up analysis of the significant interaction indicated that there was a significant effect of Session only in the WT mice (F_(11,66)_ = 2.87, *P* < 0.01), with R6/2 CAG 240 mice failing to improve with training (F_(11,66)_ = 1.65, *P* > 0.1). Genotype effects were apparent on all test days after day 2 (smallest F_(1,66)_ = 6.54, *P* < 0.02), but not on day 2 (F_(1,66)_ = 2.19, *P* > 0.2). Surprisingly, on the initial session the R6/2 CAG 240 mice performed significantly better than WT mice (F_(1,66)_ = 7.53, *P* < 0.01). The increase in discrimination performance in the WT mice was due to a more pronounced decrease in response rate as a function of training in the unreinforced than in the reinforced condition (Fig. 1B; Session x Condition interaction F_(11,33)_ = 3.34, *P* < 0.004). In contrast, R6/2 mice did not show a consistent differential change in response rate as function of condition, rather just session-to-session variability (Session main effect: F _(11,33)_ = 2.48 , *P* < 0.02; Session x Condition interaction: F_(11,33)_ = 0.57, *P* > 0.83).

In order to determine the contribution of differences in response rates between genotypes to discrimination performance, independently of changes during task acquisition, response rates from the final four days of discrimination training were analyzed separately. As shown in Fig. 1C, R6/2 CAG 240 mice tended to respond less frequently during the reinforced, and more frequently during the unreinforced conditions than did the WT mice. However, neither difference reached statistical significance, potentially due to the small sample size (reinforced: t_(6)_ = 2.19, *P* > 0.07; unreinforced: t_(6)_ = 1.33, *P*> 0.2). Clearly, the discrimination ratio is a more sensitive endpoint measure that cancels out day-to-day fluctuations in individual response rates.

**R6/2 CAG 240 mice from 9 weeks of age:**
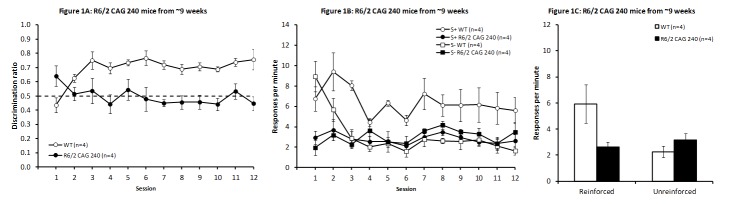
Discrimination performance in R6/2 CAG 240 mice across days of testing (A). Response rates across sessions (B) and from the mean of the final four sessions for the reinforced and unreinforced stimulus conditions (C).

**R6/2 CAG 240 F1 model**

Pre-training

Only a subset of the animals successfully passed the reinforcement criterion in this experiment sufficiently rapidly (within 15 test sessions) to be included in the discrimination training phase, leaving a substantially reduced cohort of animals in this phase. Of the initial cohort, only 9 of 16 WT mice (3 males and 6 females) and 5 of 16 R6/2 CAG 240 F1 mice (3 males and 2 females) were included in the final phase; these acquisition data are described in more detail elsewhere 17 . The animals that were included in the final phase of this study did not differ in their pre-training performance, with WT mice requiring an average of 8.6 days to reach criterion while R6/2 CAG 240 F1 mice required an average of 9.6 days (*t*_(12)_ = 0.55, *P* > 0.50).

Discrimination training

R6/2 CAG 240 F1 were significantly impaired at discrimination acquisition (see Fig. 2A), although the deficit was not as severe as it was in R6/2 CAG 240 mice described above. Analysis of discrimination ratio data with two-way ANOVA (sex and Light/Dark Condition were excluded from analysis due to the small number of R6/2 CAG 240 F1 mice in this phase of the experiment) indicated the presence of a significant interaction between Genotype and Session, along with a significant main effect of Session (smaller F_(11,132)_ = 1.88, *P* < 0.05) and a non-significant tendency towards an overall main effect of Genotype (F_(1,12)_ = 3.17, *P* < 0.071). Follow-up analysis of the significant interaction revealed that WT and R6/2 CAG 240 F1 mice significantly differed in sessions 10 – 12 (smallest F_(1,132)_ = 4.60, *P* < 0.05). The increase in discrimination performance in the WT mice was due to a higher response rate in the reinforced condition, although response rate seemed lower overall towards the end of training. Although the difference between response rates under the two conditions seemed to increase with training, the interaction of Condition and Session did not reach significance (Fig. 2B; Session and Condition main effects: F_(11,88)_ = 1.90, *P* < 0.0501 and F_(1,8)_ = 13.91, *P* < 0.006, respectively; Session x Condition interaction F_(11,88)_ = 1.43, *P* < 0.18). In contrast, R6/2 mice did not show a significant change in response rate as a function of training in either condition (Session and Condition main effects and interaction: Fs< 1.49, *P*s > 0.17).

Response rate data (Fig. 2C) from the final four days of training indicated that the R6/2 CAG 240 F1 animals did not differ from WT controls in their responding to the reinforced stimulus (t_(12)_ = 1.29, *P* > 0.2), but did respond significantly more frequently than WT control animals in the unreinforced condition (t_(12)_ = 3.38, *P* < 0.01).

**R6/2 CAG 240 F1 mice from 9 weeks of age:**
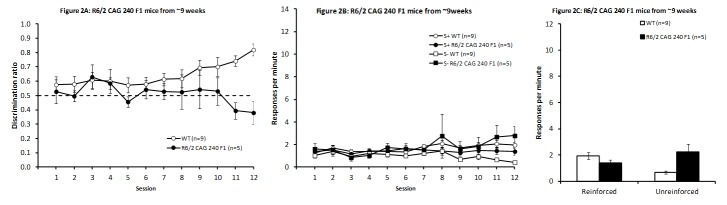
Discrimination performance in R6/2 CAG 240 F1 mice across days of testing (A). Response rates across sessions (B) and from the mean of the final four sessions for the reinforced and unreinforced stimulus conditions (C).

**BAC HD F1 model**

Discrimination training

As illustrated in Fig. 3A, no significant deficit was observed in BAC HD F1 relative to WT mice (F_(1,19)_ = 1.40, *P* > 0.2), although there was a significant main effect of Session (F_(11,209)_ = 4.74, *P* < 0.001), indicating that both BAC HD F1 and WT animals successfully learnt the task. There was no significant Genotype X Session interaction (F_(11,209)_ = 1.28, *P* > 0.2). Similarly to first R6/2 study, the increase in discrimination performance in the WT mice was due to a higher response rate in the reinforced condition, with responding in general also decreasing with training. Although there was seemingly a more robust reduction in the unreinforced than in the reinforced condition, the differential decrease was not significant, as after the first couple of sessions the response curves were rather parallel (Fig. 3B; Session and Condition main effects: F_(11,110)_ = 2.51, *P* < 0.008 and F_(1,10)_ = 17.60, *P* < 0.002, respectively; Session x Condition interaction F_(11,110)_ = 1.13, *P* > 0.34). BAC mice showed a similar pattern (Session and Condition main effects: F_(11,99)_ = 2.97, *P* < 0.002 and F_(1,9)_ = 34.26, *P* < 0.0002, respectively; Session x Condition interaction F_(11,99)_ = 0.48, *P* > 0.91).

Analysis of the last four days of training indicated no differences in response rates for either the reinforced or unreinforced conditions (larger t_(19)_ = 1.51, *P*s > 0.1; Fig. 3C).

**BAC HD F1 mice from 81 weeks:**
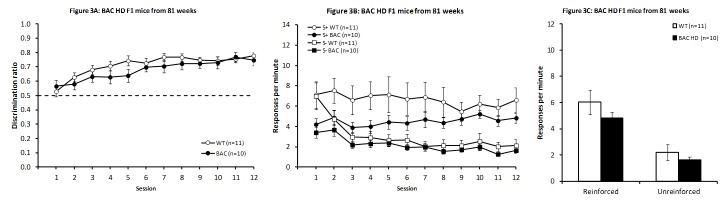
Discrimination performance in BAC HD F1 mice across days of testing (A). Response rates across sessions (B) and from the mean of the final four sessions for the reinforced and unreinforced stimulus conditions (C).

**zQ175 KI model at 74 weeks of age**

Pre-training

While all WT and zQ175 KI het animals successfully learnt to nosepoke, none of the homo animals tested here were able to acquire the nosepoke response within 15 sessions. Therefore, only WT and het animals moved on to the discrimination training stage. Amongst these mice, no differences were observed in the mean number of days required to reach criterion, with WT mice requiring an average of 3.8 days and zQ175 KI het mice requiring an average of 5.2 days (t_(8)_ = 1.34, *P* > 0.2).

Discrimination training

As shown in Fig. 4A, zQ175 KI het mice were impaired at learning the discrimination task relative to WT control mice (F_(1,8)_ = 5.67, *P* < 0.05). Nevertheless, all mice appeared to show learning, with a significant main effect of Session, (F_(11,88)_ = 10.60, *P* < 0.001), although the Genotype X Session interaction fell short of significance (F_(11,88)_ = 1.57, *P* > 0.1). As in previous experiments, the small number of mice here precluded inclusion of light/dark condition as a factor in these analyses, though no differences were apparent from inspection of the data. The increase in discrimination performance in the WT mice was due to a decrease and increase in response rate in the unreinforced and reinforced condition, respectively (Fig. 4B; Session and Condition main effects: F_(11,44)_ = 4.95, *P* < 0.0001 and F_(1,4) _= 6.30, *P* < 0.07, respectively; Session x Condition interaction F_(11,44)_ = 4.50, *P* < 0.0002). In contrast, zQ175 KI mice showed a general increase in response rate, independently of condition (Session and Condition main effects: F_(11,44)_= 4.89, *P* < 0.0001 and F_(1,4) _= 0.02, *P* > 0.88, respectively; Session x Condition interaction F_(11,44)_ = 0.68, *P* > 0.75).

During steady state responding in the last four sessions zQ175 KI het mice responded significantly more frequently than did WT controls in the unreinforced condition but not in the reinforced condition (Fig. 4C; t_(8)_ = 3.79, *P* < 0.01 and t_(8)_ = 1.22, *P*> 0.25, respectively).

**zQ175 KI mice from 74 weeks:**
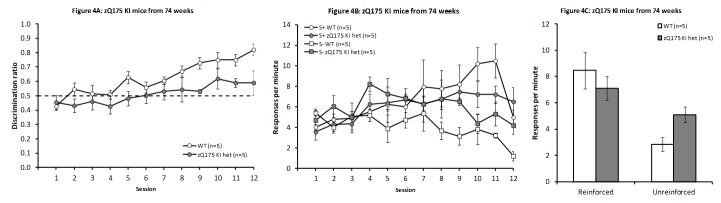
Discrimination performance across days of testing in zQ175 KI WT and het mice from 74 weeks of age (A). Response rates across sessions (B) and from the mean of the final four sessions for the reinforced and unreinforced stimulus conditions (C).

***zQ175 KI model at 54 weeks of age*** **

Pre-training

While all WT and zQ175 KI het animals successfully learnt to nosepoke, only 4 of the 10 homo mice acquired the nosepoke response rapidly enough to be included in the discrimination training period. Amongst those animals that did participate in discrimination training, there were significant differences in speed of acquisition, with WT mice requiring an average of 3.8 days to reach criterion, while zQ175 KI het mice took a mean of 6.7 days and zQ175 KI homo a mean of 11.8 days. Analysis of these data by one-way ANOVA revealed a significant overall effect of genotype (F_(2,23)_ = 14.60, *P* < 0.001), while follow-up analysis via Newman-Keuls post-hoc comparisons indicated that all three genotypes were statistically distinct from one another.

Discrimination training

The data from discrimination training are presented in Fig. 5A and indicate a pattern of deficits in the zQ175 KI het mice similar to that described in zQ175 KI mice at 74 weeks of age. One of the four homo mice included completed only 10 of the 12 training days, while one of the eleven zQ175 KI het mice completed only 11 days, with the missing data points appropriately compensated for in the SAS analysis.

The zQ175 KI homo mice appear to be even more impaired than het mice, performing below chance throughout, although this difference was not statistically reliable. Preliminary analyses were carried out with all three genotypes, with only Genotype and Session as factors, and indicated a significant effect of Genotype (F_(2,23)_ = 22.00, *P* < 0.001), but no effect of Session (F_(11,250)_ = 1.54, *P* > 0.1), and no significant interaction between Session and Genotype (F_(22,250)_ = 1.34, *P* > 0.1). Post-hoc comparisons confirmed that both zQ175 KI het and zQ175 KI homo mice significantly differed from WT animals (smaller t_(23)_ = 5.33, *P* < 0.0001), while there was a tendency towards better performance in zQ175 KI het than zQ175 KI homo animals that fell short of significance (t_(23)_ = 1.75, *P* < 0.1).

Given the small number of homo mice that reached criterion for discrimination training, further analyses were conducted with only the WT and zQ175 KI het animals, allowing more thorough analysis of these results by including Light/Dark Condition as a between-subjects factor. This analysis revealed a significant main effect of Genotype (F_(1,18)_ = 24.60, *P* < 0.001), and a significant effect of Session (F_(11,197)_ = 3.77, *P* < 0.001), indicating that while both WT and zQ175 KI het mice were able to learn the discrimination task, the WT animals performed significantly better. The interaction between Light/Dark condition and Genotype failed to reach significance (F_(1,18)_ = 3.26, *P* < 0.1), and no other effects or interactions approached significance (largest F_(1,18)_ = 1.05, remaining Fs < 1, all *P*s > 0.3).

The increase in discrimination performance in the WT mice was seemingly attributable to a higher response rate in the reinforced condition throughout training, although due to the high variability in this measure the difference did not reach significance (Fig. 5B; Session and Condition main effects: F_(11,110)_ = 1.66, *P* < 0.10 and F_(1,10)_ = 4.44, *P* < 0.07, respectively; Session x Condition interaction F_(11,110) _= 0.61, *P* > 0.81). Het zQ175 KI mice showed a slight increase in response rate, independent of condition (Session and Condition main effects: F_(11,109)_ = 2.00, *P* < 0.04 and F_(1,10)_ = 0.91, *P* > 0.36, respectively; Session x Condition interaction F_(11,109)_ = 1.62, *P* < 0.11). Homo mice did not show any effect of training or condition (F_(11,31)_ = 0.53, *P* > 0.86 and F_(1,3)_ = 4.67, *P* < 0.12, respectively; Session x Condition interaction F_(11,31)_ = 0.54, *P* > 0.86).

Examination of response rates from the last four test days (Fig. 5C) shows that zQ175 mice exhibited a tendency to respond less frequently during the reinforced condition and more frequently during the unreinforced condition when compared to WT control mice. However, there were no significant differences in response rates in either the reinforced or the unreinforced conditions (larger F_(2,23)_ = 1.36, *P*s > 0.2).

**zQ175 KI mice from 54 weeks of age:**
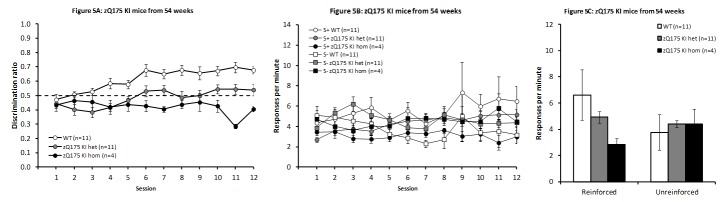
Discrimination performance across days of testing in zQ175 KI WT, het and homo mice from 54 weeks of age (A). Response rates across sessions (B) and from the mean of the final four sessions for the reinforced and unreinforced stimulus conditions (C).

***zQ175 KI model at 26 weeks of age*** **

Pre-training

All mice successfully acquired the nosepoke response, though the WT animals were significantly quicker to do so than were the zQ175 KI het mice (2.3 days vs. 4.0 days; (t_(22)_ = 2.90, *P* < 0.01).

Discrimination training

As depicted in Fig. 6A, zQ175 KI het mice were significantly impaired on this discrimination task even at a relatively early age, with testing in this experiment from 28 ± 1 weeks of age (F_(1,16)_ = 31.50, *P* < 0.001). Furthermore, there was a significant effect of Session (F_(11,176)_ = 26.40, *P* < 0.001), as in previous studies, as well as a significant interaction between Genotype and Session (F_(11,176)_ = 2.31, *P* < 0.02). Follow-up analysis of this interaction component revealed that there was significant learning in both genotypes (smaller F_(11,176)_ = 11.39, *P*s < 0.0001), while the two genotypes were significantly different at all test days except day 1, where there was no genotype difference (F < 1.00, *P* > 0.5), and day 6, where the difference fell just short of significance (F_(1,176)_ = 3.23, *P* < 0.08; smallest remaining F_(1,176)_ = 7.49, all *P*s < 0.01).

This dataset also revealed a significant overall effect of Light/Dark Condition (F_(1,16)_ = 5.18, *P* < 0.05, which interacted significantly with Session (F_(11,176)_ = 2.35, *P* < 0.02), although Light/Dark Condition did not significantly interact with Genotype (all Fs < 1.00, all *P*s > 0.5). As is illustrated in Fig. 6B, this interaction resulted from significantly improved performance early in training in animals reinforced for nosepoking the light stimulus, with significant effects of light/dark condition on days 3, 4 and 5 (smallest F_(1,176)_ = 8.41, *P*s < 0.01), along with a marginal difference on day 6 (F_(1,176)_ = 2.80, *P* < 0.1), but no differences were observed later in training or on days 1 and 2 (largest remaining F_(1,176)_ = 1.82, all *P*s > 0.15). Significant overall effects of Session were observed in both light/dark conditions (smaller F_(11,176)_ = 11.90, *P*s < 0.001). No further effects or interactions reached significance, though there was a tendency towards an overall interaction of sex with Light/Dark Condition (F_(1,16)_ = 3.76, *P* < 0.08, largest remaining F_(1,16)_ = 1.10, largest remaining F_(11,176)_ = 1.02, all *P*s > 0.3).

No further effects or interactions reached significance, though there was a tendency towards an overall interaction of sex with light/dark condition [F_(1,16)_ = 3.76, *P* < 0.08, largest remaining F_(1,16)_ = 1.10, largest remaining F_(11,176)_ = 1.02, all *P*s > 0.3].

The increase in discrimination performance in the WT mice was due to higher response rate in the reinforced condition throughout, except in the first session (Fig. 6C), although this initial effect was not captured by a significant Session x Condition interaction (Session and Condition main effects: F_(11,121)_ = 10.71, *P* < 0.0001 and F_(1,11)_ = 106.53, *P* < 0.0001, respectively; Session x Condition interaction F_(11,121)_ = 1.50, *P* < 0.15). Het zQ175 KI mice showed a similar but seemingly less pronounced differential response to the reinforcer condition (Session and Condition main effects: F_(11,121)_ = 5.98, *P* < 0.0001 and F_(1,11)_ = 10.42, *P* < 0.008, respectively; Session x Condition interaction F_(11,121)_ = 1.56, *P* < 0.13).

Furthermore, consistent with an overall impairment in discrimination performance in the zQ175 mice, evaluation of response rates during the final four sessions (Fig. 6D) indicated reduced rates of responding in the zQ175 KI het animals in the reinforced condition (t_(22)_ = 2.88, *P* < 0.01), although they responded marginally more frequently than WT mice in the unreinforced condition, however, this effect fell short of significance (t_(22)_ = 1.99, *P*< 0.06).

**zQ175 KI mice from 26 weeks of age:**
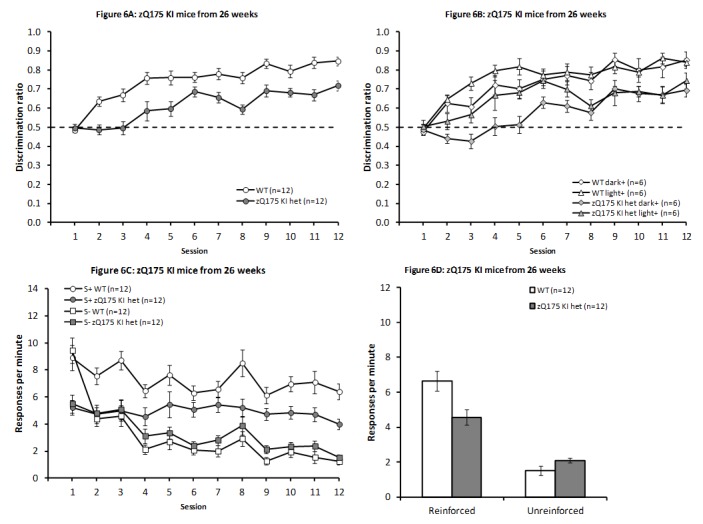
Discrimination performance across days of testing in zQ175 KI from 26 weeks of age. Discrimination data are presented both collapsed across light/dark condition (A) and separately (B). Response rates across sessions (C) and from the mean of the final four sessions for the reinforced and unreinforced stimulus conditions (D).

## Discussion

Recent studies have indicated that R6/2 mice have deficits in a timing task, the peak procedure 16 that could be explained in terms of a deficit in response inhibition. Other studies have focused on different aspects of cognitive function: KI HD models have been shown to have deficits in motor learning in a serial implicit learning task 23. HD mouse models have also been studied in choice discrimination tasks, including swim tank, touchscreen and set shifting paradigms, which have demonstrated deficits in several models 13 ^,^ 14 ^,^ 15 ^,^ 21 ^,^ 24 ^,^ 25. The present experiments reveal clear deficits in discrimination ratios in a modified Go/No-go visual discrimination task in both the R6/2 CAG 240 transgenic and the zQ175 KI mouse models of HD, though not in the BAC HD F1 transgenic model. Overall, this assay seems a promising and relatively simple method to measure some aspects of executive dysfunction in these HD mouse models.

Although mice were not strictly required to withhold responding during the unreinforced period, it is noted that the initial training involved reinforcing responses to one stimulus followed by a second phase in which a second unreinforced stimulus was introduced, therefore implying that a reduction in the response rate during the last phase of training required inhibition of responding. Moreover, in this sense, the presently-employed task closely resembles other modified Go/No-go procedures that have been utilized to assess response inhibition in rodents 26 ^,^ 27 ^,^ 28. Crucially, all Go/No-go tasks involve a level of discrimination as different cues are associated with differing reinforcement schedules. In turn, not all discrimination tasks necessarily have a response inhibition component.

HD mice responded at least as frequently as their respective WT controls during the unreinforced period, significantly more so in the cases of the R6/2 CAG240 F1 and 74 week old zQ175 mice. Indeed, the present results suggest that impairments in discrimination performance in HD mice were driven in part by excessive response rates during the unreinforced period, consistent with deficits in the ability to inhibit inappropriate responding. Future studies of discrimination performance and response inhibition may utilize a procedure in which mice are reinforced for withholding responding during presentation of the negative discriminative stimulus, allowing for stricter evaluation of stimulus control of behavior.

In the present task, reinforcement was delivered to mice following responding during the period in which the positive discriminative stimulus was present. It is therefore possible that in addition to the visual stimulus, delivery of the reinforcer itself functioned as a discriminative stimulus, raising the question of whether responding was strictly under control of the visual stimulus or under control of a composite of reinforcer delivery coupled with the visual stimulus. In any case, it is important to note that the precise nature of the stimulus controlling behavior is unlikely to explain observed deficits in discrimination performance.

The nature of the underlying deficits in discrimination learning and response inhibition likely stem from reduced ability in the HD mice to control a learned response, with the HD mice more prone than controls to continue making responses even when reinforcement is not available. Importantly, response inhibition can be described as consisting of two underlying processes, namely “action restraint” and “action cancellation” 29. Under this construct, modified Go/No-go procedures such as the one employed presently are thought to evaluate action restraint, such that subjects must decide whether or not to proceed with responding prior to initiating a response. In contrast, tasks that assess action cancellation, such as stop-signal reaction time tasks 30, require that subjects terminate a response after it has been initiated (see 31 for review). It is possible that the presently-reported deficits in action restraint are one aspect of more broadly construed deficits in response inhibition that also include an action cancellation component. To this end, there is presently a pronounced lack of published reports assessing stop-signal reaction time in mice (see 32 for review); however, it would be of great translational value to evaluate mouse models of HD using such a task, including assessment of such deficits at an age when more global inhibition deficits such as those assessed in this task are still not apparent. A concern, however, is the extent to which known bradykinesia in HD mice can confound stop-signal tasks in which reaction time is a crucial component.

Keeping in line with specific impairments in action restraint, the present results may serve to explain, at least in part, deficits in behavioral flexibility, including reversal learning 13 ^,^ 24 and attentional set shifting 14 ^,^ 15, which have been reported in mouse models of HD. In terms of translation to the clinic, tasks that assess behavioral flexibility are analogous to attentional ‘switching’ tasks like the Wisconsin Card Sorting Task, which involve several parallel executive processes, including attention, working memory and response inhibition. Such tasks require participants to inhibit implementation of a previously learned rule and have been shown to reveal deficits in HD patients 11 ^,^ 33. In sum, the inability to inhibit inappropriate responding is consistent with, and may partly underlie deficits in behavioral flexibility which are documented in humans with prodromal HD as well as mouse models of the disease. Furthermore, similar tasks specifically measuring response inhibition have been used to assess attentional and inhibitory processes in different diseases, with HD patients presenting with robust deficits in these tasks 34 ^,^ 35.

There is potential for concern in evaluating mouse models with known motor deficits (see 2 ^,^ 19 ^,^ 20), inasmuch such deficits might confound performance in cognitive assays. In the present experiments it is worth noting that there is a specific risk associated with calculating discrimination ratios that, in a hypothetical situation where one group of animals cannot respond as frequently as another, a difference in discrimination ratio could be driven solely by reduced response rate in the reinforced condition, rather than inability to learn the discrimination task, per se. The present data cannot be explained entirely in this manner, however, since the primary contributor to the poor discrimination ratios recorded in both R6/2 CAG 240 and zQ175 KI het mice is that they are prone to respond as frequently as, if not more, than WT controls in the unreinforced condition, rather than that they are unable to respond as frequently in the reinforced condition. We show that most of the improvements in discrimination ratios (in WT mice) were driven by appropriate response rate decreases in the unreinforced condition as a function of training, rather than increases in the reinforced condition, thus failure of mutant mice to do so cannot be attributed to motor deficits without being extremely unparsimonious.

Another potential issue with this type of food-reinforced experiment in comparing different mouse models is variable levels of motivation to consume the food reward, such that apparently reduced learning might simply reflect disinterest or lack of hunger. As much as possible, these studies have been designed to minimize these risks, employing a low effort nosepoke response on a rich reinforcement schedule (VI5) to encourage responding in the discrimination task along with a highly-valued reinforcer (evaporated milk). Simple consumption tests carried out on separate cohorts of mice maintained in the same manner as the current test animals have revealed that these HD mouse models and WT controls consume similar quantities of the reinforcer milk when presented with 30 min consumption tests, suggesting that the food restriction procedures employed lead to comparable degrees of motivation in these mouse lines. Moreover, as noted previously, response rates in the reinforced conditions were generally similar across genotypes in these experiments, with the discrimination deficits being driven by a relatively high rate of responding in the unreinforced condition. Taken together, the present results are not consistent with an explanation based, at least solely, on reduced hedonic motivation.

As has been described elsewhere 18, operant tasks offer a valuable tool for assessment of transgenic animals generally, and HD mouse models specifically, providing a reasonably high throughput, largely automated, and sensitive set of tools to assess subtle cognitive and motor phenotypes in these animals. The present experiments describe a relatively simple task that reveals deficits in two different HD mouse models based on different genetic constructs, indicating that these deficits are not specific to the particular mouse line tested. Notably, a distinct advantage of this paradigm over others is that it requires only a relatively basic operant apparatus with a single nosepoke recess and a food magazine. The total time required for this training is not excessive, with an experiment potentially completed within four weeks from initial food restriction to completion of discrimination training. This factor is of particular relevance when testing mouse models of neurodegenerative disease that display a progressive increase in cognitive and motor symptom severity. Moreover, due to their relative simplicity, go/no-go tests are amenable to translating to the clinical setting and can be relatively easily implemented for testing HD patients. This latter point would be of especially high value, with the potential to streamline the translation of novel therapies for cognitive impairment in patients with HD.

## Competing Interests

The authors have read the journal’s policy and have the following interests to declare. PsychoGenics conducted the research through a fee-for-service agreement for CHDI Foundation. David Howland is employed by CHDI Management, Inc. as an advisor to CHDI Foundation, Inc. Stephen Oakeshott, Russell Port, Jane Sutphen, Jason Berger, Judy Watson-Johnson, Sylvie Ramboz, Dani Brunner and Andrew Farrar are or were employed by PsychoGenics. There are no patents, products in development or marketed products to declare. We fully adhere to all the PLOS policies on sharing data and materials.
